# The rewarding nature of provocation-focused rumination in women with borderline personality disorder: a preliminary fMRI investigation

**DOI:** 10.1186/s40479-018-0079-7

**Published:** 2018-01-16

**Authors:** Jessica R. Peters, David S. Chester, Erin C. Walsh, C. Nathan DeWall, Ruth A. Baer

**Affiliations:** 1 Department of Psychiatry and Human Behavior, Alpert Medical School of Brown University and Rhode Island Hospital, 700 Butler Drive, Providence, RI 02906 USA; 2 Department of Psychology, Virginia Commonwealth University, Richmond, VA USA; 3 Department of Psychiatry, University of North Carolina at Chapel Hill School of Medicine, Chapel Hill, NC USA; 4 Department of Psychology, University of Kentucky, Lexington, KY USA

**Keywords:** Borderline personality disorder, Anger, Rumination, Reward, fMRI, Criticism, Nucleus accumbens, Insula, Provocation

## Abstract

**Background:**

Understanding why individuals with borderline personality disorder (BPD) ruminate on prior provocations, despite its negative outcomes, is crucial to improving interventions. Provocation-focused rumination may be rewarding in the short term by amplifying anger and producing feelings of justification, validation, and increased energy, while reducing self-directed negative affect. If provocation-focused rumination is utilized regularly as a rewarding emotion regulation strategy, it could result in increased activation in reward-related neural regions. The present pilot study examined neural correlates of provocation-focused rumination, relative to other forms of thought, in BPD.

**Method:**

Functional magnetic resonance imaging (fMRI) was utilized to examine this theory in a pilot study of women diagnosed with BPD (*n* = 13) and healthy controls (*n* = 16). All participants received highly critical feedback on a previously written essay in the scanner, followed by prompts to engage in provocation-focused, self-focused, and neutral thought.

**Results:**

Whole-brain analyses showed that in response to the provocation, participants with BPD (compared to controls) demonstrated increased activation in the ventrolateral prefrontal cortex (PFC). BPD participants also showed greater activation in the dorsomedial PFC during provocation-focused rumination (relative to neutral-focus). Subsequent ROI analyses revealed that provocation-focused rumination (compared to neutral-focus) increased activation in the nucleus accumbens for the BPD group only.

**Conclusions:**

These findings, while preliminary due to the small sample size and limitations of the protocol, provide initial data consistent with the proposed neurobiological mechanism promoting provocation-focused rumination in BPD. Directions for further research are discussed.

## Background

Borderline personality disorder (BPD) is characterized by affective instability, identity disturbances, problems in interpersonal relationships, intense anger, and self-destructive impulsivity [1]. Rumination, defined as repetitive, passive, unconstructive thinking about negative emotions and problems [2], may contribute to amplifying and maintaining these patterns of negative affect and dysfunctional behavior. Although many people falsely assume that extended thinking about problems will lead to insight and solutions [3, 4], rumination intensifies negative affect and reduces problem-solving ability. Anger rumination, in which individuals focus on angry moods and prior provocations, is particularly associated with BPD features [5, 6] and predicts characteristics of BPD, such as anger, displaced aggression, and cognitive distortions [7–9]. While individuals with BPD vary in the extent to which they anger ruminate, these robust findings suggest it is a common behavior in this population. To develop more effective treatments, it is crucial to understand why individuals with BPD tend to engage in rumination on provocations and anger despite its negative outcomes.

### Function of anger rumination in BPD

One proposed function of anger rumination is avoidance of more aversive emotions and cognitions [10]. Shame proneness is endemic to BPD [11–13], as is pronounced rejection sensitivity [14–16]. Ruminating on anger may reduce this internally directed negative affect, by focusing instead on external causes for distress, such as unfair situations and deplorable behavior of others [13]. The resulting amplified anger contributes to the aggression and interpersonal problems typical of BPD, potentially increasing risk of future social rejection and feelings of shame. Consistent with this theory, self-reported anger rumination and anger have been shown to mediate the relationship between shame-proneness and BPD features in a student sample [13], and individuals diagnosed with BPD have been shown to react strongly to rejection cues with rage [17].

Anger is typically conceptualized as a negative emotion, but it also has immediate positive outcomes, such as increased energy and feelings of justification. Most negative emotions induce avoidant behavior; however, like positive affect, anger increases approach motivation [18]. Therefore, anger rumination may not only dampen BPD individuals’ self-directed negative affect (negative reinforcement), but also provide feelings of validation, empowerment, and pleasure (positive reinforcement). These positive immediate effects may be particularly reinforcing given that interpersonal experiences typically considered rewarding, such as praise from others, fail to produce positive affect for individuals with BPD and may actually be evaluated negatively [19]. If the theory proposed is accurate, individuals with BPD should experience altered neural activity in reward networks during provocation-focused rumination, following increased neural reactivity to critical feedback.

### Neural correlates of reactivity to criticism

In healthy individuals, social exclusion activates neural regions involved in affective distress, including the dorsal anterior cingulate cortex (dACC) and the anterior insula (AI), as well as the ventrolateral prefrontal cortex (VLPFC) [20, 21]. Activation of this system may function as a neural alarm that promotes recognition of and responses to the event [21]. Alterations in these neural regions have been implicated in reactivity to rejection and interpersonal interactions in BPD. In one study comparing neural reactivity to rejection, inclusion, and neutral conditions during a behavioral task in individuals with BPD and healthy controls, the rejection condition elicited relatively greater dACC activation compared to inclusion and neutral conditions within each group as expected; however, the BPD group also demonstrated a main effect of higher levels of dACC and dorsomedial prefrontal cortex (DMPFC) activation relative to controls across all task conditions [22]. These findings suggest that, while activation in the dACC may increase in response to rejection for both individuals with BPD and controls, for those with BPD, this may occur in conjunction with a generally heightened level of activation in this system when evaluating social situations.

The VLPFC, which co-activates with the dACC and insula in response to social exclusion, is associated with regulation of negative emotions and inhibition of psychological pain [20, 23, 24]. Amplifying activation in the VLPFC prior to and during a social exclusion paradigm attenuated emotional reactivity [25]. Conversely, inhibiting the VLPFC following social exclusion amplified the normative negative emotional response [26]. Together, these findings suggest a key role for the VLPFC in regulating affective reactivity to social rejection.

### Neural correlates of anger rumination

Several neural regions have been specifically linked to anger rumination in a non-clinical sample. Denson et al. [27] employed an interpersonal provocation manipulation where an experimenter was rude and implied participants were not intelligent enough to follow directions. Then, during fMRI scanning, the participants received sets of prompts (counter-balanced in order) to engage in various forms of thought: provocation-focused (e.g., “Think about how you have interacted with the experimenter up to this point”), self-focused (e.g., “Think about why you react the way you do”), and neutral-focused (e.g., “Think about the layout of the local post-office”) [27]. Compared to neutral-focus, both provocation- and self-focused conditions involved greater recruitment of regions related to anger and affective responses to social rejection (dACC), emotion regulation (LPFC), arousal (thalamus, insula), and self-referential thought (dorsomedial prefrontal cortex; DMPFC). Activation of the DMPFC and right anterior insula across both rumination conditions, compared to the neutral-focused condition, correlated with self-reported state rumination and trait-level displaced aggression. The study did not obtain any whole-brain findings for reward-related regions in this non-clinical sample, nor were any reward-related ROIs hypothesized about or examined.

### Neural correlates of reward

Positive reinforcement activates the ventral striatum, specifically the nucleus accumbens (NAcc), a central node in learning, motivation, and reward circuitry see [28] for review. Recruitment of the NAcc has most reliably been associated with experiences of reward and subjective pleasure [29–31], occurring in response to a range of appetitive cues and pleasurable activities including both naturally occurring rewards (e.g., money, food, orgasm) and drugs of abuse [32]. Additionally, pleasant mental imagery also been selectively activates the NAcc and the MPFC, with the degree of NAcc activation correlated with the extent of pleasure endorsed [33].

While individuals with addiction behaviors, such as substance abuse, tend to demonstrate baseline hypoactivity of reward networks, these regions, including the NAcc, show increased activation during anticipation of relevant appetitive cues [34]. This NAcc sensitization to rewarding stimuli creates a learned motivational response facilitating addiction even in the absence of withdrawal symptoms [35], suggesting this process could also facilitate non-drug habits. Consistent with this, NAcc sensitization has been demonstrated during the anticipation of eating [36], planning of food binges [37], and decision-making about retaliatory aggressive behavior [38] for individuals with maladaptive levels of these behaviors in daily life.

BPD-specific striatal alterations may be linked to difficulties with emotion regulation. Striatal regions functioned similarly in BPD patients and controls in response to monetary rewards in emotionally neutral contexts; however, in the context of emotional images, BPD patients demonstrated reduced reward differentiation and less deactivation of reward circuitry following cue exposure [39]. One possibility is that emotional reactivity disrupts reward systems for BPD patients [39]. Alternatively, for emotionally reactive individuals, emotional cues could have greater potency as reward or punishment than small amounts of money. These findings hint at the possibility that emotionally evocative stimuli and processes, such as anger rumination, may function as BPD-relevant appetitive cues.

### Clarifying the function of provocation-focused rumination in BPD

The present pilot study utilized fMRI to compare blood oxygen level dependent (BOLD) signal changes in specific brain regions among participants with BPD and healthy controls across the experiences of interpersonal provocation and ruminative responding. In response to provocation, participants with BPD (vs. controls) were expected to demonstrate higher activation in brain regions associated with reactivity to social rejection (AI, dACC, VLPFC). All participants were expected to demonstrate greater activation in regions previously associated with anger rumination (dACC, DMPFC) during subsequent provocation-focused thought compared to neutral-focused thought; however, this effect was expected to be greater for participants with BPD. Participants with BPD (vs. controls) were predicted to experience greater activation in brain regions associated with reward and pleasure (NAcc) during provocation-focused thought.

## Methods

### Participants

Participants (*n* = 31) were right-handed women who were at least 18 years old. Thirteen of them met the DSM-IV criteria for BPD. The other 18 were age-matched healthy controls. All participants were screened for suitability for MRI research. Individuals were excluded who reported neurological pathology or injury, developmental disorders, prior or current problematic substance use, psychotic symptoms, and claustrophobia (determined through interview with the participant about their lifetime history of diagnoses, injuries, substance use, and discomfort in enclosed spaces, as well as several questions assessing delusions and hallucinations)—these screeners were conducted on the phone and then repeated in-person. Control participants were required to meet no criteria for BPD and to have never received any other psychological diagnosis or treatment and not to be using psychoactive medication. Of the BPD group, 11 were not on any psychoactive substances at the time of the study, and 2 were taking SSRI medication. Only one member of the BPD group had recently begun receiving dialectical behavior therapy; most other BPD group participants reported prior lifetime experience with psychotherapy (not BPD-specific), however were not currently in therapy for a range of reasons (e.g., previous therapy was not helpful, finances). All participants were offered low-cost psychotherapy referral options following the experiment as part of the debriefing process.

Recruitment occurred from contacts with local clinics and psychotherapists, craigslist advertisements, study fliers, and introductory psychology classes at a large, public university. Participants received either $100 for participating or course credit. Advertisements for the BPD group did not mention BPD specifically, given that prior BPD diagnosis was not required; instead, the fliers read, “*You may be eligible to participate if experience intense emotions and difficulties in relationships*.” Participants who responded to the advertisement and expressed interest were then administered a phone screener for BPD symptoms. For the BPD group, those who endorsed 5 or more criteria on a brief phone screen based on the complete BPD diagnostic interview (*N* = 22) were invited to participate; only those who met criteria for BPD during the in-person diagnostic interview (*N* = 17) were asked to return for the scanning session. Of those, 14 returned for the scan session. Two of the final BPD group participants were recruited from the psychology classes, one from an outpatient clinic, and eleven from the general community.

### Measures

#### Structured clinical interview for the DSM-IV Axis II disorders (SCID-II; [40])

The SCID-II is a standardized, semi-structured, clinician administered interview for diagnosing DSM-IV Axis II mental disorders. The BPD section only of the SCID-II was administered by an advanced doctoral candidate in clinical psychology and interviews and scoring reviewed with a licensed clinical psychologist.

#### Personality assessment inventory borderline features scale (PAI-BOR; [41])

The PAI-BOR is a well-validated measure of four aspects of BPD pathology: affective instability, identity problems, negative relationships, and self-harm. Raw scores on the total scale above 37 (*T* > 70) are considered to be in the clinical range and predict BPD-specific dysfunction in clinical, community, and student samples [41, 42], while raw scores below 18 (*T* < 30) represent absent to minimal BPD-related symptoms. In the present study, PAI-BOR total score demonstrated good to excellent internal consistency (α = .84–.96).

#### Anger rumination scale (ARS; [43])

The ARS has 19 items assessing the tendency to focus attention on angry moods, recall past anger episodes, and think about the causes and consequence of anger episodes. Responses range from 1 (“almost never”) to 4 (“almost always”). The ARS total score demonstrated excellent internal consistency in the present study (α = .96).

#### Center for Epidemiological Studies—Depression (CES-D; [44])

The CES-D is a 20-item inventory of depressive symptoms. The CES-D asks participants to rate their mood, thoughts, and behavior during the previous week on a 4-point Likert scale, ranging from 0 (“rarely or none of the time”) to 3 (“most or all of the time”). In the present study, the CES-D demonstrated excellent internal consistency (α = .94).

#### PTSD checklist—Civilian version (PCL-C; [45])

The PCL-C is a 17-item questionnaire that asks participants to rate the extent they have been bothered by PTSD symptoms over the past month. Responses range from 1 (“not at all”) to 5 (“extremely”). In the present study, the PCL demonstrated excellent internal consistency (α = .95).

### Procedure

#### Preliminary screening

A phone screen was administered to all potential participants including the diagnostic and MRI safety screeners. Participants were also administered a risk assessment; this was repeated in person for individuals who enrolled in the study and to ensure safety at points throughout the study. Individuals were excluded for current urges to engage in harm to self or others; participants in the BPD group could endorse lifetime self-harm or suicidality. These phone interviews and all subsequent clinical interviews and assessments were conducted by an advanced clinical psychology doctoral student.

#### Assessment session

Participants (*N* = 43) completed self-report measures of BPD symptoms, and the SCID-II for BPD was then administered. Any participants who did not meet inclusion criteria (no BPD criteria met for the control group; at least five BPD criteria fully endorsed for the BPD group) were excluded from the second study session.

#### Scanning session

Participants (*N* = 31) completed the scanning session, which took place between 2 and 10 days after the assessment visit.

##### Essay-writing paradigm

Participants were asked to write a short essay about a time in which someone else angered them. In accordance with a previously validated provocation paradigm [46], they were told that a research assistant would evaluate it on several key criteria and that this feedback would be provided while they are in the MRI scanner. Each participant’s essay was given the same harsh criticism, regardless of what they had written (see Scanning Procedure).

##### Scanning procedure

Each MRI scanning session included two experimental tasks that were completed while fMRI was acquired. After a high-resolution anatomical scan was completed, participants were removed from the scanner.

*Provocation Task*. The provocation manipulation was created by combining a previously-used fMRI provocation procedure [27] with an insulting essay feedback paradigm used in behavioral research, that has previously been demonstrated to produce a robust increase in anger [46]. This procedure was chosen over the one previously used with directed rumination task (where the experimenter personally delivered the provocation), in order to maintain the alliance between the experimenter and participant in the event of safety concerns, given the clinical sample. The task was divided into three blocks (pre-feedback baseline, feedback, post-feedback baseline) [27]. In the first, pre-feedback block, participants passively viewed a fixation cross to capture baseline neural activity (120 s). Next, participants viewed a prompt to “Get Ready to View Your Essay Feedback” (5 s). Then, participants viewed a series of five ratings of various characteristics of their essay (10 s per rating; e.g., “clarity of expression”, “writing style”) that were preprogrammed to be insulting (1/7–3/7 points) as well as a total score (10/35; 10s). All participants received the same ratings. Then, participants viewed their reviewer’s ‘comments’ on their essay for 10 s, which was: “One of the worst essays I have EVER read!” Finally, participants viewed another fixation cross to model post-feedback baseline neural activity (120 s).

*Directed Rumination Task (DRT).* To assess neural activity specific to angry rumination (i.e., repetitive thoughts about the provocation participants had just experienced), participants completed a shortened version of a previously validated paradigm in which participants are directed to ruminate about three topics in succession: the prior provocation (provocation-focus condition), themselves (self-focused condition), and a neutral topic (neutral-focus condition) [27]. The three block-types were presented in counter-balanced order across participants, within groups. The task was implemented in a block design. In each block, participants viewed a series of 6 statements (15 s per statement; 90s per block), that instructed participants what content to ruminate about.^1^ During provocation-focused blocks, participants read rumination prompts with statements instructing them to engage in anger rumination, reflecting on the provoking incident encountered earlier in the study (e.g., “Think about how you have been treated” “Think about whether your treatment was unfair or unreasonable”). During self-focused blocks, participants read statements instructing individuals to think about themselves (e.g., “Think about what kind of a person you are.” “Think about why you respond to others the way you do.”). During neutral-focused blocks, participants read prompts with statements instructing individuals to reflect on neutral statements unrelated to the study (e.g., “Think about the layout of the local post office”, ‘Think about a bus driving down the street”). Between blocks of the DRT, participants were given a 30 s rest period with a fixation cross, followed by a 5-s prompt to get ready for the next set of statements.

After exiting the scanner, participants were told of the deception involved in the writing task and provocation.

### Data acquisition and analyses

#### fMRI data acquisition

All images were collected on a 3.0 T Siemens Magnetom Trio scanner using a Siemens 32-channel head coil. Functional echo planar images were acquired with a T2*-weighted gradient echo sequence with a 3D shim applied before functional data acquisition (matrix size = 64 × 64, field of view = 224 mm, echo time = 28 ms, repetition time = 2.5 s, slice thickness = 3.5 mm, 40 interleaved axial slices, flip angle = 90°). These parameters allowed for whole-brain coverage with 3.5 mm cubic voxels. A high-resolution, coplanar T1-weighted image was also acquired from each participant so that functional data could be registered to native anatomical space and then normalized to the Montreal Neurological Institute (MNI) atlas space (1mm^3^ isotropic voxel size, echo time = 2.56 ms, repetition time = 1.69 s, flip angle = 12°).

#### fMRI preprocessing

All preprocessing and statistical analyses were conducted using FSL (Oxford Center for Functional Magnetic Resonance Imaging [FMRIB] [47, 48]). Functional volumes were reconstructed from k-space and the reconstructed functional volumes were corrected for head movement to the median volume using MCFLIRT [49], corrected for interleaved slice-timing skew using temporal sync interpolation, pre-whitened using FILM, and spatially-smoothed with a 5 mm full-width-half-maximum Gaussian kernel. To remove drifts within sessions, a high-pass filter was applied (200 s cutoff). Non-brain structures were stripped from functional and anatomical volumes using FSL’s Brain Extraction Tool [50].

#### fMRI data analyses

We modeled within-subjects, between-subjects and between-groups (BPD vs. control) variance in brain activation utilizing a 2-stage summary statistics approach to multi-level modeling via FSL. An initial fixed-effects general linear model (GLM) modeled event-related responses for each run of each participant using a canonical double-gamma hemodynamic response function with a temporal derivative. All six motion parameters were modeled as nuisance regressors for all analyses. For the Provocation Task, pre-feedback baseline, feedback, and post-feedback baseline blocks were each separately modeled as regressors in the model, with pre-block instructions modeled as a nuisance regressor. Within the Provocation Task, we contrasted feedback with pre-feedback baseline (feedback > pre-feedback baseline), to assess the effects of critical feedback on activation. For the DRT, provocation-focus, self-focus, and neutral-focus blocks were modeled as regressors in the first-level GLM. Pre-block instructions were modeled as a nuisance regressor and fixation trials were left unmodeled to serve as an implicit baseline. Within the DRT task, we separately contrasted provocation-focus with both self-focus and neutral-focus blocks, as well as self-focus contrasted with neutral-focus, to assess activation specific to each of those conditions.

##### Whole-brain analysis

To model these contrasts at the group level, we performed top-level, mixed-effects GLM analyses, which created group average maps for contrasts of interest and allowed us to contrast BPD and control groups. For each lower-level contrast (e.g., provocation-focused rumination > self-focused rumination), group-level *Z* (Gaussianized T/F) statistic images were created and then thresholded using clusters determined by *Z* > 2.3 and a (familywise error corrected) cluster significance threshold of *p* < .05. In addition to these group-level aggregate analyses, we created contrast maps that compared BPD participants to controls using the same thresholding procedures previously described. Cluster thresholding was applied both across the whole brain.

##### Regions of interest analysis

We also employed an a priori region of interest (ROI) approach for the DRT task to investigate the effects of rumination on ROIs implicated in anger rumination and reward processing. Four ROIs in the DMPFC (left superior DMPFC, right superior DMPFC, left medial DMPFC, and right medial DMPFC) and two in the dACC (right dACC, left dACC) were based on an activation clusters found in previous research on activation in these regions during anger rumination, compared to neural thought [27]. Each ROI was constructed using an 8 mm-radius sphere around each cluster’s peak voxel. Given that no published studies to date have directly examined the effects of provocation-related rumination on reward-related brain regions, we also examined ROIs in order to provide critical preliminary tests of our central hypothesis. Region of interest (ROI) masks were constructed for the right and left NAcc from the Wake Forest Pickatlas toolkit [51]. For each task condition, parameter estimates were extracted (in units of percent signal change) and averaged across all voxels of each ROI. Parameter estimates were then analyzed in SPSS via Group (BPD, Control) x Condition (Provocation-, Self-, Neutral- Focus) ANOVA, with Bonferroni corrections employed for post-hoc contrasts to control for familywise error rates.

#### Power

Power was estimated for comparisons across the DRT, the primary analyses of interest. For between and within-subject effects in the GLM, power ranged from 11 to 14% for small effects (*d* = .2), 42–66% for medium effects (*d* = .5), and 80–98% for large effects (*d* = .8), based on Cohen’s effect sizes [52]. The study was a relatively small, preliminary exploration of a novel theory; accordingly, it was not powered to detect smaller effects.

## Results

### Data screening

Data were screened for outliers on all measures. One participant was removed from analyses due to values greater than 3 SD above the mean for the entire sample for activation of the right and bilateral NAcc during the provocation > neutral contrast during the DRT. One control participant was removed prior to analyses due to endorsement of one of the DSM BPD criteria during the debriefing session. The final sample analyzed included 28 participants (BPD group = 13; control group = 16).

### Demographics and self-report

Groups did not significantly differ by age (see Table 1), race (χ^2^ = .76, *p* = .69), or education level (χ^2^ = 4.12, *p* = .13). Accordingly, these demographic variables were not controlled for in subsequent analyses. To confirm validity of SCID-II diagnoses, PAI-BOR scores for the BPD group were compared to the control group (see Table 1 for group comparisons of all self-report variables). As expected, the BPD group reported significantly higher levels of BPD symptoms. The control group endorsed a mean level of PAI-BOR total scores in the low symptoms category, with no control participants reporting above average symptom levels, whereas the BPD group’s mean was clinically elevated, with 85% reporting clinically elevated symptoms and two participants endorsing above average levels. Also consistent with previous studies, the BPD group reported generally engaging in a significantly higher level of anger rumination than the control group. The BPD group also reported a significantly greater level of symptoms of depression and PTSD than the controls. Group means for the CES-D were similar to previous studies comparing women with BPD to healthy controls [11], with 1 (6%) control participant and 10 (77%) BPD participants endorsing symptom levels consistent with elevated risk of depression. For the PCL-C, 1 (6%) control participant and 8 (62%) BPD participants endorsed symptom levels above the screening thresholds for elevated risk for PTSD.

**Table 1:** Differences between control and BPD groups on self-report measures of BPD symptoms, anger rumination, and age (*N* = 29)

	HC Mean (SD)	BPD Mean (SD)	t	*p*-value
PAI-BOR	9.88 (5.02)	44.23 (8.75)	13.28	<.001
ARS	1.26 (.20)	2.55 (.37)	11.32^a^	<.001
CES-D	7.44 (6.65)	28.23 (9.27)	7.03	<.001
PCL	23.69 (8.65)	49.31 (13.21)	6.29	<.001
Age	21.81 (4.02)	21.23 (3.30)	−.42	.58

*PAI-BOR* Personality Assessment Inventory-Borderline Features Subscale, *ARS* Anger Rumination Scale, *CES-D* Center for Epidemiological Studies Depression Scale, *PCL* Post-Traumatic Stress Disorder Checklist

*t*-tests conducted with equal variances assumed except where denoted by (^a^)

### Imaging results

#### Provocation task

**Fig. 1:**
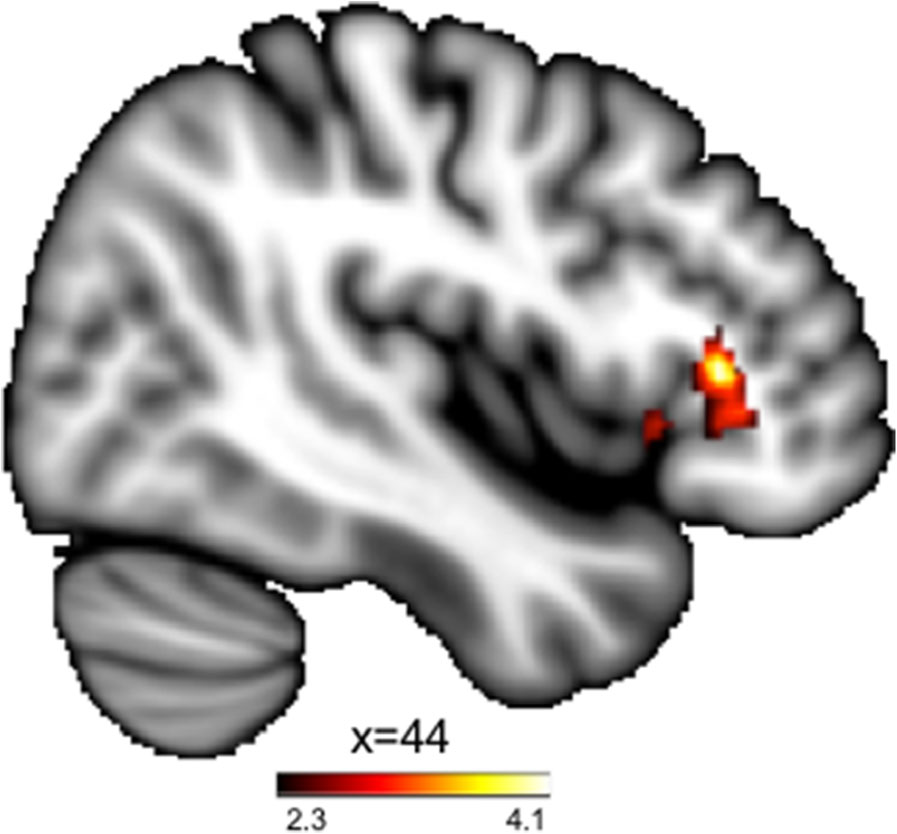
During the Provocation Task, greater activation of the right VLPFC was observed among BPD participants (compared to Controls)

In the whole-brain analyses, the BPD > Control between-group contrast revealed a cluster of increased activation in the VLPFC (peak coordinates: inferior frontal gyrus as defined by Harvard-Oxford cortical structural probabilistic atlas) that extends into the orbitofrontal cortex, operculum, and anterior insula, in response to the negative essay feedback (Fig. 1, feedback > pre-feedback baseline contrast; Table 2).

**Table 2:** Regions of activation in the BPD group vs controls

Region	MNI coordinates			*k*	Peak z-score
	X	Y	Z		
*Critical Essay Feedback:* Provocation paradigm > Baseline					
Right VLPFC	44	30	6	397	4.14
*Directed Rumination Task:* Provocation-focused thought > Neutral-focused thought					
DMPFC	2	48	38	696	4.46

#### Directed rumination task

##### Whole-brain analysis

**Fig. 2:**
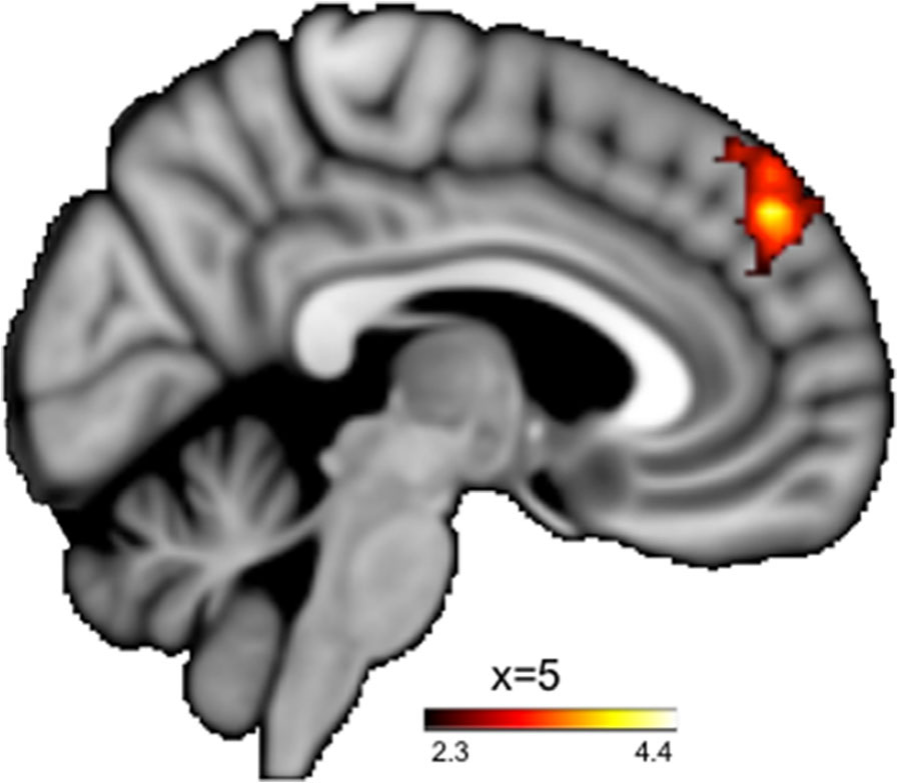
During the Directed Rumination Task, BPD participants (compared to Controls) showed increased activation of the DMPFC provocation-focus (vs. neutral-focus)

Whole-brain analyses revealed a significant between-group difference in neural activation on the Directed Rumination Task. The BPD > Control contrast revealed greater activation in midline DMPFC during provocation-focused rumination (as compared to the neutral condition; Fig. 2; Table 2). No significant effects of BPD diagnosis were found on self-focused rumination (as compared to the neutral condition).

##### ROI analysis

To reduce familywise error, the DMPFC ROIs were combined into a single index averaging across the ROIs, demonstrating high internal consistency within each DRT contrast (α > .88). The DACC ROIs were similarly combined into a single index for each contrast (α > .93). Right and left NAcc ROIs were also combined into an index representing bilateral NAcc activation for each contrast (α > .76) .^2^

GLM analyses demonstrated no group by condition interaction on DMPFC activation (F [2, 54] = 1.47, *p* = .24), but a significant main effect of DRT condition on this DMPFC index (F [2, 54] = 4.96, *p* = .010, *d* = .86). Post-hoc contrasts revealed significant greater activation in the provocation condition compared to neutral (*t* = 4.10, *p* < .001, *d* = .76) and the self-condition compared to neutral (*t* = 2.73, *p* = .011, *d* = .50), with no significant contrast between the provocation and self-focused condition (*t* = .82, *p* = .42). A similar pattern of findings emerged for the dACC ROI, with no group by condition effects (F [2, 54] = .32, *p* = .73), but a significant main effect of DRT condition (F [2, 54] = 3.86, *p* = .027, *d* = .76), with post-hoc testing revealing significantly greater activation in the provocation-focused compared to neutral condition (*t* = 3.05, *p* = .005, *d* = .57), with no significant contrasts for the self-focused condition (*p* > .22).

Second, we examined bilateral NAcc activation across the conditions of the DRT. GLMs estimating NAcc activation from condition, group, and condition by group interaction were modeled. When all three DRT conditions were included in the models, neither a significant group by condition interaction effect on the bilateral NAcc was observed (F [2, 54] = 2.56, *p* = .087) nor a main effect of condition alone was observed (F [2, 54] = 2.19, *p* = .12).

**Fig. 3:**
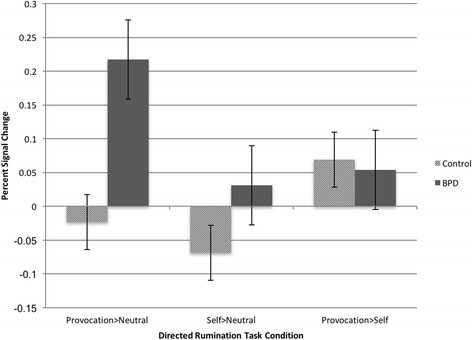
Nucleus accumbens percent signal change for task contrasts by diagnostic group (BPD vs Control) during the Directed Rumination Task

Given the DMPFC and dACC ROI findings suggesting that the self-focused condition was not well differentiated from the provocation condition, exploratory GLMs for the NAcc were estimated containing only the provocation-focused and neutral-focused conditions, to test the contrast of primary interest. A significant group by condition interaction, with a large effect size, was found for NAcc right activation (F [1, 27] = 6.38, *p* = .018, *d* = 0.94). Probing this interaction demonstrates that, as hypothesized, for individuals with BPD, the provocation-focus condition, compared to neutral focus, led to increased activation in the NAcc (t [12]= 2.27, *p* = .018, *d* = 0.76), whereas for controls, no significant differences between these two conditions were observed (t [15] = − .41, *p* = .69; see Fig. 3 for DRT condition contrasts in bilateral NAcc activation by group).

As a post-hoc exploratory analysis, associations between right NAcc activation contrasts and self-reported BPD features, anger rumination, depression symptoms, and PTSD symptoms were also computed, using Spearman-Rank correlations given non-normal distribution of variables (see Table 3). The provocation > neutral contrast demonstrated significant, moderate associations with the PAI-BOR features of self-harm, negative relationships, and affective instability. Associations with self-reported anger rumination, depression, PTSD symptoms, and identity disturbance were not significant, although effect sizes observed were moderate across all variables. No significant associations were observed between the self > neutral contrast nor the provocation > self contrast and any of the self-report variables. Intercorrelations between all self-report measures were generally extremely high (*r*s = .75–.86), with associations with the PAI-BOR self-harm subscale and others slightly lower (*r* = .64–72).

**Table 3:** Spearman-rank correlations between bilateral nucleus accumbens activation contrasts during the Directed Rumination Task and self-report measures of BPD features, anger rumination, and symptoms of depression and PTSD (*N* = 29)

Condition contrast	ARS	PAI-BOR AI	PAI-BOR ID	PAI-BOR NR	PAI-BOR SH	CES-D	PCL
Provocation > Neutral	.31	.37^a^	.33	.46^a^	.46^a^	.37	.32
Self > Neutral	.24	.18	.31	.25	.18	.34	.22
Provocation > Self	.06	.13	−.02	.14	.21	.01	.07

^a^*p* <.05

*ARS* Anger Rumination Scale, *PAI-BOR* Personality Assessment Inventory Borderline Personality Disorder Scale, *AI* Affective Instability, *ID* Identity Disturbance, *NR* Negative Relationships, *SH* Self-Harm, *CES-D* Center for Epidemiological Studies Depression Scale, *PCL* Post-Traumatic Stress Disorder Checklist

## Discussion

The results of this pilot study demonstrated mixed support for our hypotheses; however, they do provide some preliminary data consistent with the hypothesis that provocation-focused rumination may selectively activate neural regions associated with reward for individuals with BPD. While engaging in provocation-focused thought, relative to neutral-focus, all participants demonstrated greater activation in most of the regions previously associated with anger rumination and self-referential thought (DMPFC, dACC) [27], suggesting both groups engaged in the task; however, greater relative activation in regions of the DMPFC in provocation-focused thought in individuals with BPD, compared to controls, perhaps reflects greater intensity of engagement with the provocation stimuli for the BPD group.

ROI analyses showed differences in neural activation in regions associated with reward responsiveness during the DRT. Although the hypothesized group by condition interaction across all three directed rumination task conditions was not significant for NAcc activation, exploratory contrasts between the provocation- and neutral-focused conditions only revealed that while controls demonstrated no differences in reward-activation (NAcc ROI activation) between the neutral-focused and provocation-focused conditions, the provocation-focused condition produced significantly more activation in the right NAcc than neutral-focused thought for the BPD group. Furthermore, this increased NAcc activation during provocation-focused vs neutral-thought was correlated across the sample with self-report measures of BPD features of affective instability, negative relationships and self-destructive behavior.

Combined with the finding of greater activation in the right VLPFC and parts of the AI for the BPD group during the prior critical feedback, these findings are consistent with the theory that individuals with BPD are more reactive to criticism, possibly including greater efforts to regulate their emotional responses, and that they may find the experience of ruminating about the provocation more rewarding than healthy controls. This sequence of reactions could contribute to the well-established tendency of individuals with BPD to endorse high trait levels of both internally directed negative affect (shame) and externally directed negative affect (anger), aggression, and impulsive behavior. However, this interpretation of the data is limited by its use of reverse inference, and it is possible that these patterns of neural activation may reflect different or additional neural processes. For example, activation of the ventral striatum (including the NAcc) could reflect emotional enhancement of learning [53]. Further work combining neuroimaging with additional, task-based methods to measure emotional reactivity and reward responses would provide more robust tests of this theory that rely less on reverse inference. The interpretation is also limited by the lack of affect ratings or other non-neural data of participant reactivity to the inductions; without these, it is difficult to know whether there were group differences in affect, attribution, or interpretation of the task and precisely what form of affect was elicited during criticism and the rumination prompts and how these may have varied across participants or groups. The present analyses utilized ROIs previously linked to anger rumination and anger in the study that developed this task; however, it cannot be confirmed that these ROIs are linked to the same subjective experiences in the present sample. Further research is needed to establish whether these findings are specific to rumination following increased subjective experiences of anger; if confirmed, that would provide a stronger and more specific link between NAcc activation during anger-related rumination in BPD and better support for the theory in question.

As hypothesized, the BPD group demonstrated greater recruitment of the VLPFC, as well portions of the AI, when receiving critical feedback than controls. Activation of the VLPFC occurs in emotion regulation efforts, including those that result in increased negative affect [23]. The peak area of greater activation for the BPD group was in the inferior frontal gyrus, a region that may play a key role in efforts to engage in response inhibition [54]. Its activation may represent detection of a salient response regardless of eventual behavioral action [54]. Findings of greater reactivity in parts of the AI also are consistent with previous work on reactivity to distress in BPD, with a meta-analysis of negative emotion processing concluding that patients with BPD demonstrate hyper-reactivity in the right insular cortex [55]. Thus, in the present study, these findings may represent greater salience of the criticism, greater perception of the criticism as distressing, and/or greater effort required by the BPD group to process and attempt to regulate their emotional responses to the criticism.

Contrary to hypotheses, there were no significant differences between groups in dACC activation during this task. Some previous findings demonstrate deactivation of the ACC in BPD in negative emotion inductions, and the present results are consistent with a theory that the strong response in the insula to distress may, for some individuals with BPD, lead to suppression of ACC activation and thus facilitate dissociative experiences [56]. Another issue to consider is that individuals with BPD may be more reactive to stimuli conforming to BPD-specific themes (e.g., rejection and abandonment) [57, 58]. The critical feedback may have been experienced as both notice of having done poorly on the task and also as potentially unfair, but not as an incident of social rejection. Different effects in both the feedback phase and following ruminative thought might be achieved if a more explicitly interpersonal critique had been levied, such as critical feedback regarding the person’s potential as a friend after meeting them. Future work should incorporate these to determine more precisely the nature of the group differences in responses to the manipulations. A limitation of these findings is that the provocation > baseline contrast includes both provocation and other processes (including reading, social cognition, and self-evaluation); future studies should examine these effects using an active baseline control involving similar processes, such as reading neutral evaluations.

Similar to previous research [27], in the ROI analyses, the self-focus condition did not produce significantly different levels of NAcc or DACC activation from the other conditions for either group, and did not differ from the provocation condition in DMPFC activation. For nonclinical individuals, none of these forms of thought differentially activated the NAcc, whereas for individuals with BPD, focusing on the self may fall at an indistinguishable midpoint between neutral-focused and provocation-focused. One possible explanation for this finding is that the self-focused prompts may also invoke components of anger, particularly following an angering experience for the BPD group. Future research utilizing other more specific affective thought inductions, such as a depressive-focus condition or worry-focus, may clarify the extent to which the neural responses demonstrated in this study are specific to anger.

While the present study demonstrated differences between women with BPD and healthy controls, it is not clear the extent to which these effects are specific to BPD. SCID-II interviews were only conducted to evaluate BPD criteria, and therefore the extent of co-morbid other disorders is not known, although their existence likely. The BPD sample endorsed elevated scores on depression and PTSD screeners; however, these values are similar to those found in other BPD clinical samples [11], and screeners best distinguish between individuals with diagnoses and healthy controls but do not function optimally within other clinical samples [59]. Both depressive and PTSD symptom endorsement were highly correlated with BPD symptoms in the present study, as to be expected in this sample of only individuals with BPD and healthy controls. NAcc activation during provocation-focused (vs neutral) thought was significantly associated with BPD symptoms only; however, given the small sample size and potential restriction of range issues, the specificity of these findings should still be interpreted cautiously. Given that excluding commonly co-morbid diagnoses (e.g., depressive disorders) can limit the external validity of a BPD sample, extending this work with clinical comparison groups (such as individuals with depression and/or anxiety disorders) would best clarify the specificity of these findings to BPD. It is possible these findings may be attributable to comorbid psychopathology or to a broader transdiagnostic process relevant to multiple diagnoses, including BPD. This study is also limited by a small sample size; follow-up studies with larger samples could explore these theories with greater power. Using larger samples would also allow for testing of potential moderators of these effects within the BPD group, including co-morbid diagnoses and other individual differences, which is especially important given the heterogeneous nature of the diagnosis. The current study also used female participants; future work should examine these effects in men, as well as determining whether sex may moderate the effects.

## Conclusions

These findings have potential clinical implications for the treatment of BPD. If provocation-focused rumination following interpersonal criticism is a rewarding experience for these women, that may explain why they do it despite the long-term negative consequences. It also may make it difficult for them to stop engaging in provocation-focused rumination or to be motivated to try to stop, even if they are aware of its detrimental effects. This reward-sensitization could also foster other addictive tendencies. Bidirectional cross-sensitization has been demonstrated between substances and naturally occurring rewards, such as food and sex [60–62], with sensitization to one stimuli increasing responses to the other due to common neural mechanisms [63]. Individuals with BPD demonstrate elevated rates of impulsive behaviors such as substance abuse, binge-eating, and risky sexual behavior [1]; early sensitization to provocation-focused rumination-related reward could contribute to these vulnerabilities.

Interventions targeting anger rumination may need to utilize techniques designed for other behaviors that are rewarding in the short term, such as substance abuse. Motivational interviewing [64], for example, may help individuals acknowledge the effects of their behavior and increase their readiness to make changes. Current approaches to BPD treatment, such as dialectical behavior therapy DBT; [65, 66], teach mindfulness skills for increasing awareness of thoughts and emotions and skills for managing urges and tolerating distress without engaging in risky behaviors. Applying these specifically to anger rumination may help patients to identify when they feel distress from interpersonal interactions, to recognize when they are engaging in anger rumination, and to substitute less harmful behaviors for managing those emotions. Increasing acceptance of initial emotional reactivity to criticism may also reduce the value of the reward of externalizing blame. Cognitive emotion regulation strategies have been shown to affect striatal responses to reward cues in a non-clinical sample [67]. Further research should examine whether interventions attenuate the NAcc activation found in the present study during anger rumination for individuals with BPD or whether any strategies may help with self-control despite sustained NAcc activation.

## Abbreviations

ACC: anterior cingulate cortex
AI: anterior insula
ARS: Anger Rumination Scale
BPD: borderline personality disorder
CES-D: Center for Epidemiological Studies Depression Scale
dACC: dorsal anterior cingulate cortex
DMPFC: dorsomedial prefrontal cortex
DRT: Directed Rumination Task
fMRI: functional magnetic resonance imaging
NAcc: nucleus accumbens
PAI-BOR: Personality Assessment Inventory-Borderline Scale
PCL: Post-Traumatic Stress Disorder Checklist
PET: positron emission topography
ROI: region of interest
SCID-II: Structured Clinical Interview for the DSM-IV Axis II
VLPFC: ventrolateral prefrontal cortex

## Footnotes

1 The first two participants completed a longer version of this task, in which blocks consisted of 12 statements (15 s per statement; 180 s per block).

2 When analyzed separately, ANOVA results with original ROI estimates all demonstrate the same patterns of significance as those with the bilateral indices created.
